# Supplementary material for: Dynamical Behavior of the Human Ferroportin Homologue from Bdellovibrio bacteriovorus: Insight into the Ligand Recognition Mechanism
Source: Int J Mol Sci. 2020 Sep 16;21(18):6785. doi: 10.3390/ijms21186785 (PMC7555787; doi:10.3390/ijms21186785)
Supplement: Supplementary file 1 [file ijms-21-06785-s001.pdf]

International Journal of *Molecular Sciences*

Article

Dynamical behavior of the human ferroportin homologue from *Bdellovibrio bacteriovorus*. Insight into the ligand recognition mechanism.

Valentina Tortosa[1], Maria Carmela Bonaccorsi di Patti[2], Federico Iacovelli[3], Andrea Pasquadibisceglie[1], Mattia Falconi[3], Giovanni Musci[4], Fabio Polticelli[1,5*]

[1]Department of Sciences, Roma Tre University, 00146 Rome, Italy; valentina.tortosa@uniroma3.it (V.T.); andrea.pasquadibisceglie@uniroma3.it (A.P.)
[2]Department of Biochemical Sciences, Sapienza University of Roma, 00185 Rome, Italy; mariacarmela.bonaccorsi@uniroma1.it (M.C.B.P.)
[3]Department of Biology, University of Rome Tor Vergata, 00133 Rome, Italy; federico.iacovelli@uniroma2.it (F.I.); falconi@uniroma2.it (M.F.)
[4]Department Biosciences and Territory, University of Molise, 86090 Pesche, Italy; giovanni.musci@unimol.it (G.M.)
[5]National Institute of Nuclear Physics, Roma Tre Section, 00146 Rome, Italy
*Correspondence: fabio.polticelli@uniroma3.it; Tel.: +39-06-5733-6362

Supplementary Materials

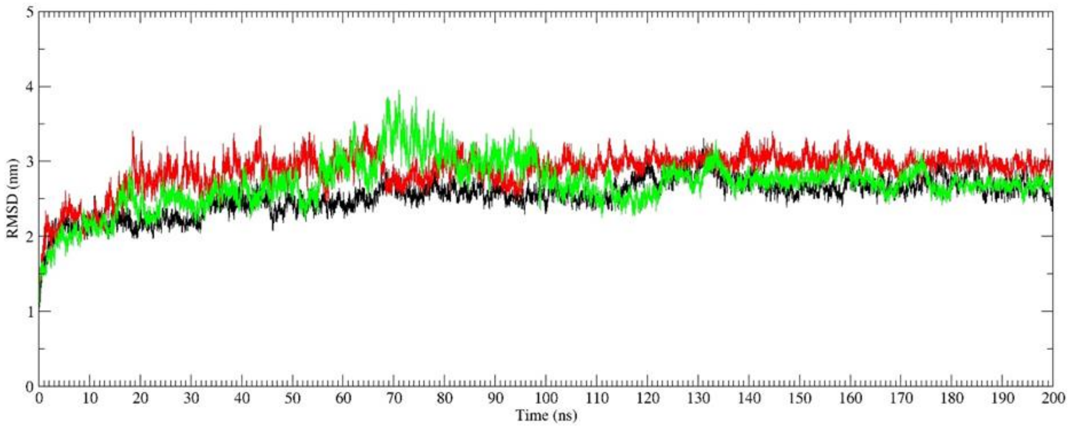

Figure S1. Root mean-square deviation (RMSD) values calculated on protein heavy atoms for the three simulation replicas of the inward-facing conformation as a function of simulation time.

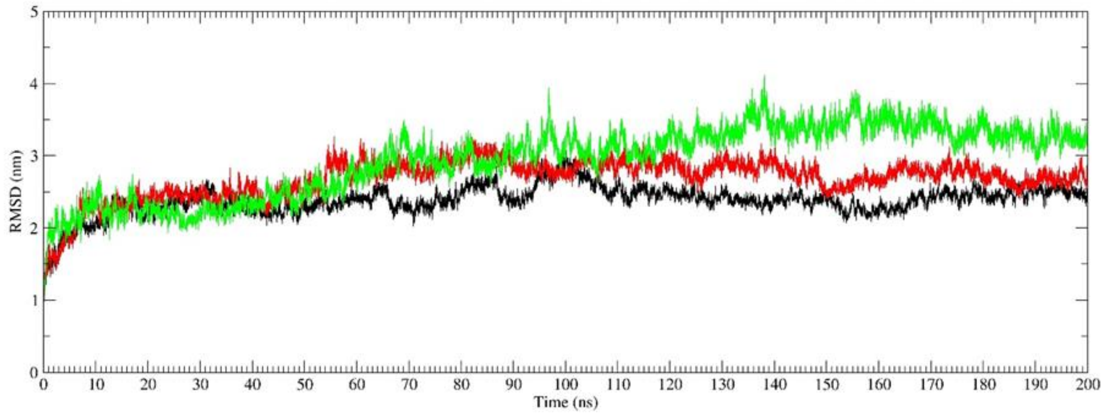

Figure S2. RMSD values calculated on protein heavy atoms for the three simulation replicas of the outward-facing conformation as a function of the simulation time.

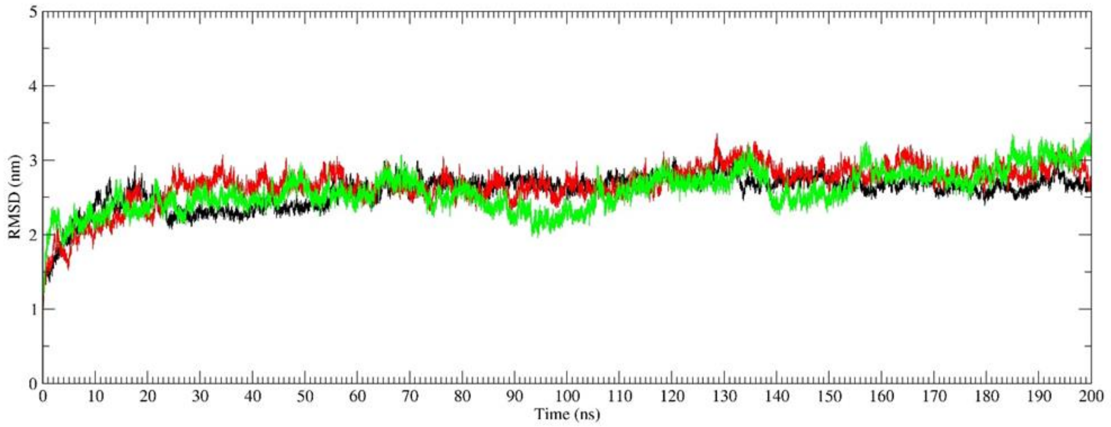

Figure S3. RMSD values calculated on protein heavy atoms for the three simulation replicas of the Fe_WT as a function of the simulation time.

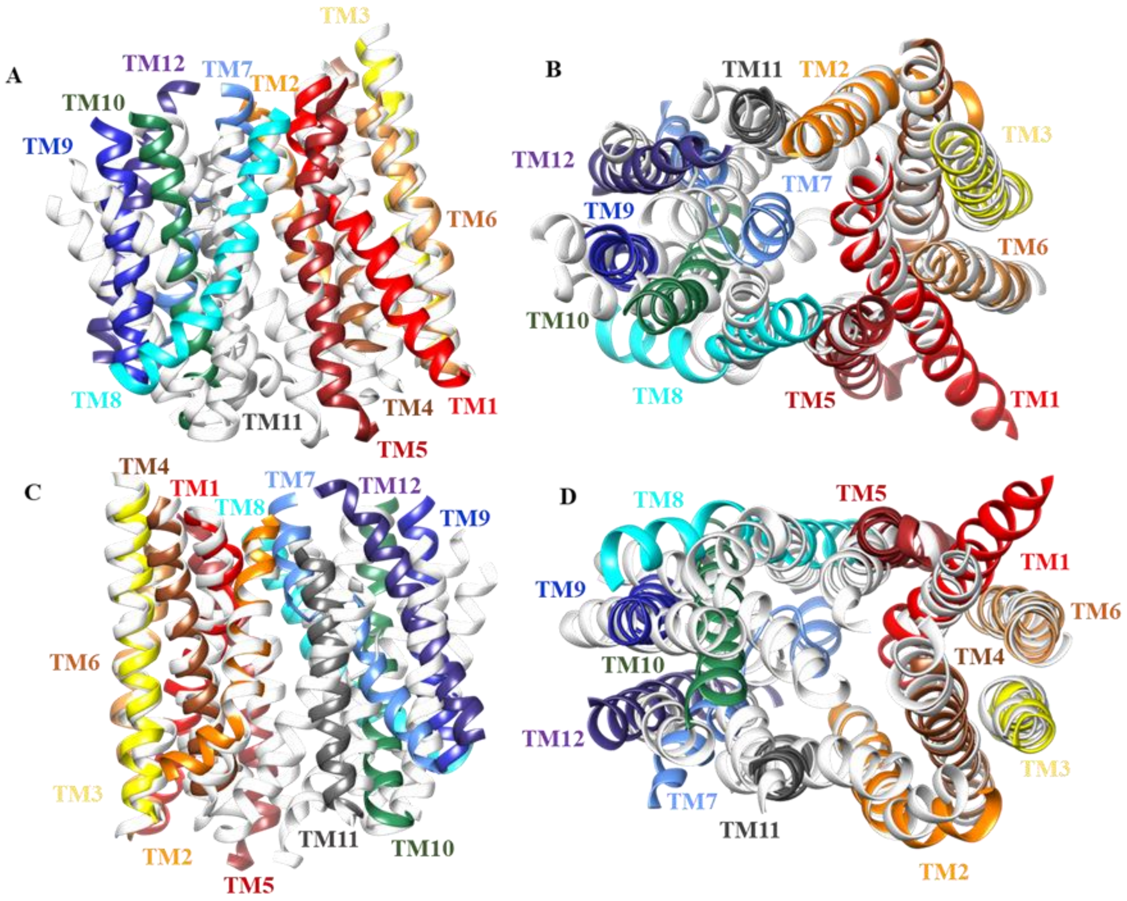

Figure S4 Structural comparison of the inward-facing (rainbow helices) and outward-facing states (white helices), (A front view; B front view rotated of 180°; C extracellular view; D intracellular view).

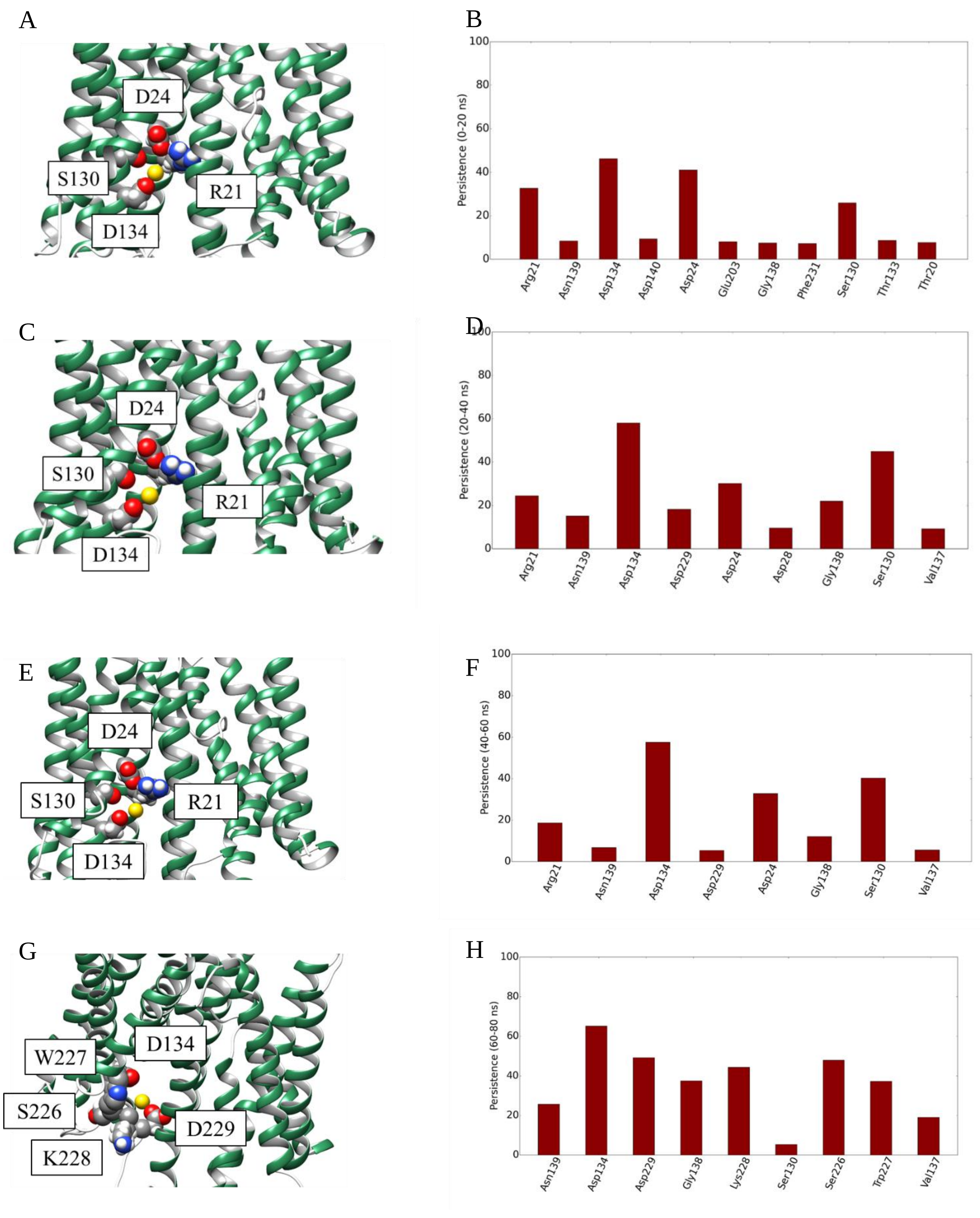
A
D24
S130
R21
D134
B
Persistence (0-20 ns)
100
80
60
40
20
0
Arg21
Asn139
Asp134
Asp140
Asp24
Glu203
Gly138
Phe231
Ser130
Thr133
Thr20
C
D24
S130
R21
D134
D
Persistence (20-40 ns)
100
80
60
40
20
0
Arg21
Asn139
Asp134
Asp229
Asp24
Asp28
Gly138
Ser130
Val137
E
D24
S130
R21
D134
F
Persistence (40-60 ns)
100
80
60
40
20
0
Arg21
Asn139
Asp134
Asp229
Asp24
Gly138
Ser130
Val137
G
W227
D134
S226
D229
K228
H
Persistence (60-80 ns)
100
80
60
40
20
0
Asn139
Asp134
Asp229
Gly138
Lys228
Ser130
Ser226
Trp227
Val137

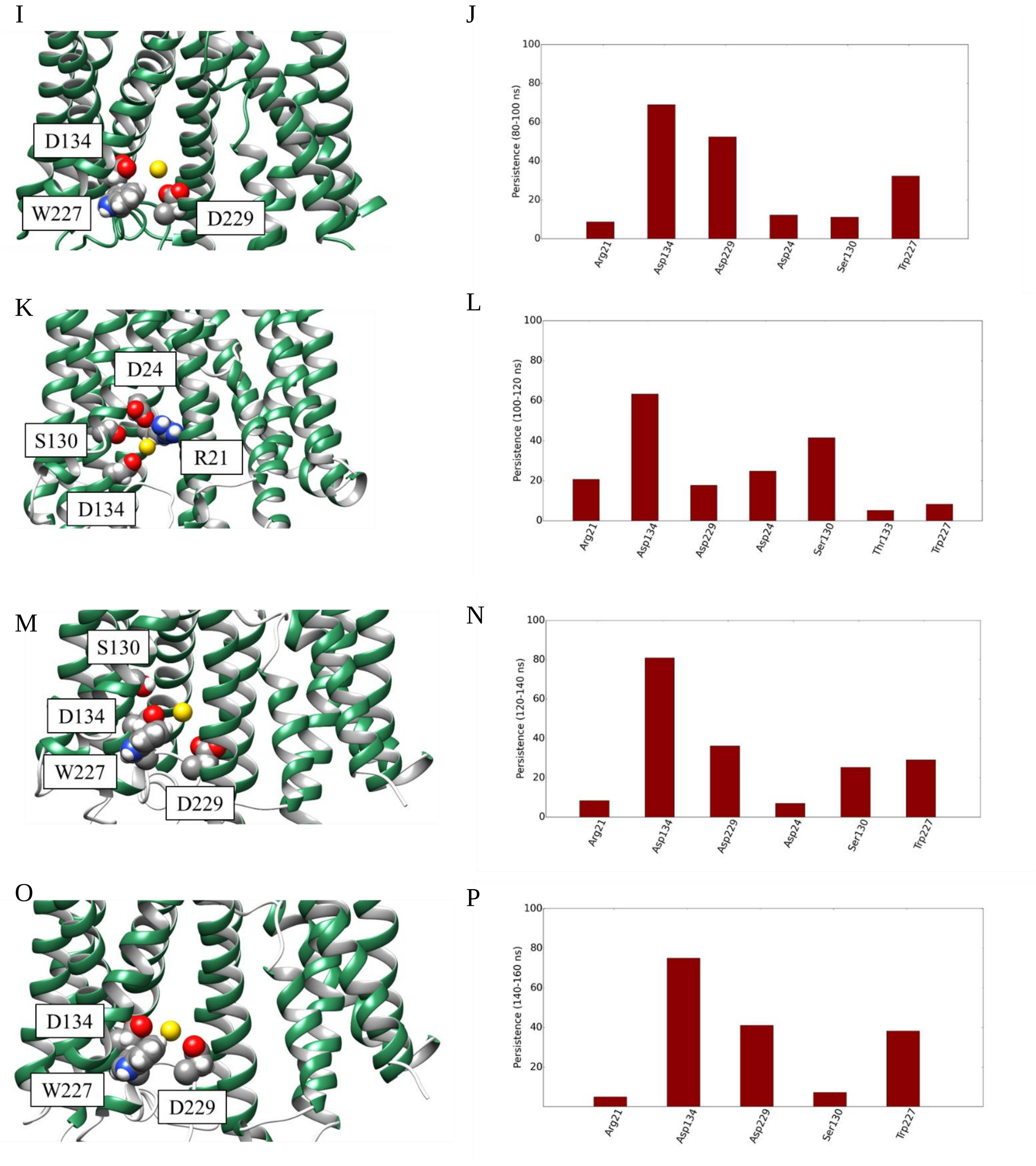
I
D134
W227
D229
J
Persistence (80-100 ns)
Arg21
Asp134
Asp229
Asp24
Ser130
Trp227
K
D24
S130
R21
D134
L
Persistence (100-120 ns)
Arg21
Asp134
Asp229
Asp24
Ser130
Thr133
Trp227
M
S130
D134
W227
D229
N
Persistence (120-140 ns)
Arg21
Asp134
Asp229
Asp24
Ser130
Trp227
O
D134
W227
D229
P
Persistence (140-160 ns)
Arg21
Asp134
Asp229
Ser130
Trp227

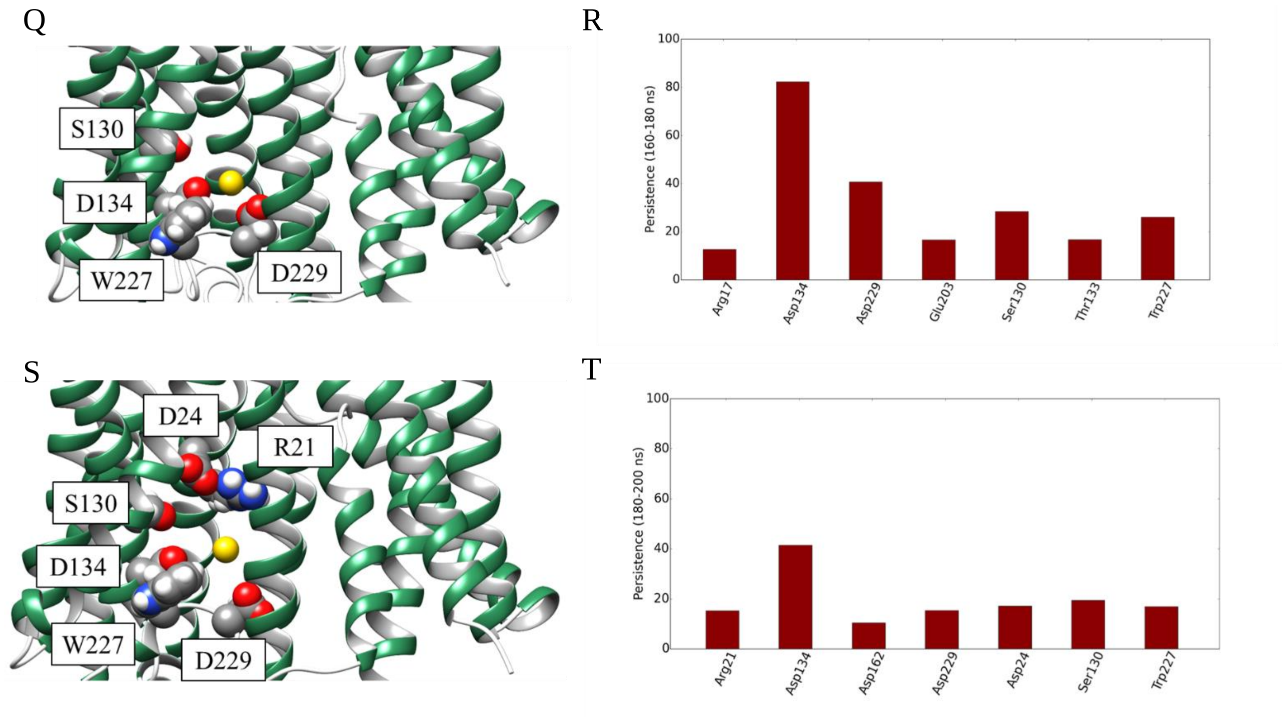

Figure S5. Structural representation of the main iron interacting residues (panels **A,C,E,G,I,K,M,O,Q,S**), and iron persistence histogram (in percent of the simulation time) (panels **B,D,F,H,J,L,N,P,R,T**), for every 20 ns interval. The persistence has been calculated by an in-house written script. Residues are colored by atom type; iron is colored in yellow.

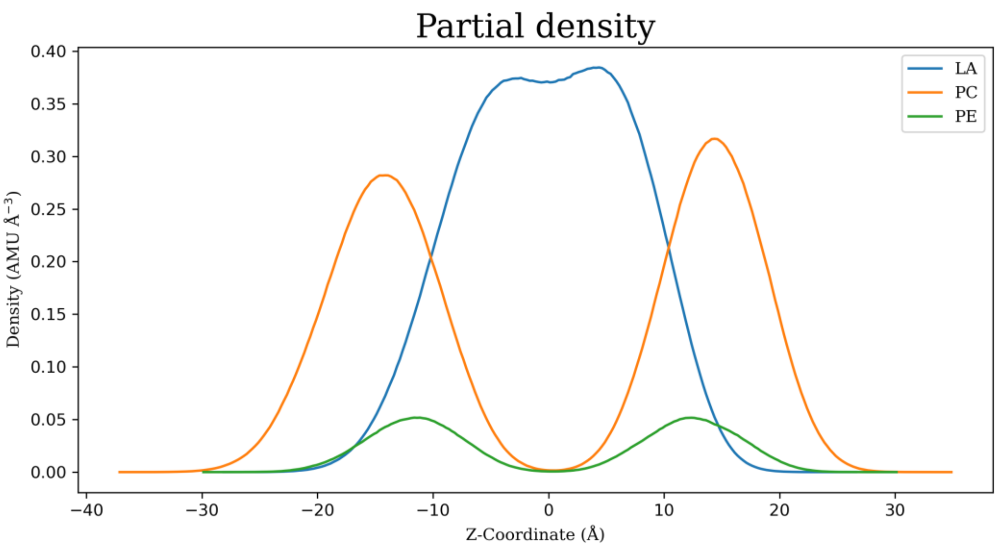

Figure S6. Density calculated for the lipids headgroups (phosphatidylcoline, PC; phosphatidylethanolamine, PE) and for the acyl chains (lauroyl, LA) of the membrane, averaged along the trajectory and the surface area of the inward-facing BdFpn structure.

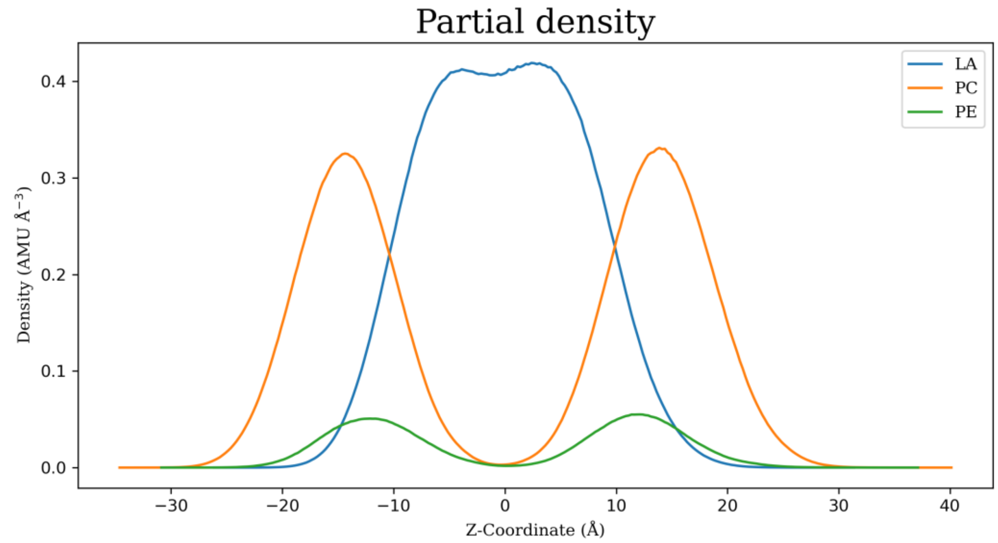

Figure S7. Density calculated for the lipids headgroups (phosphatidylcoline, PC; phosphatidylethanolamine, PE) and for the acyl chains (lauroyl, LA) of the membrane, averaged along the trajectory and the surface area of the outward-facing BdFpn structure.

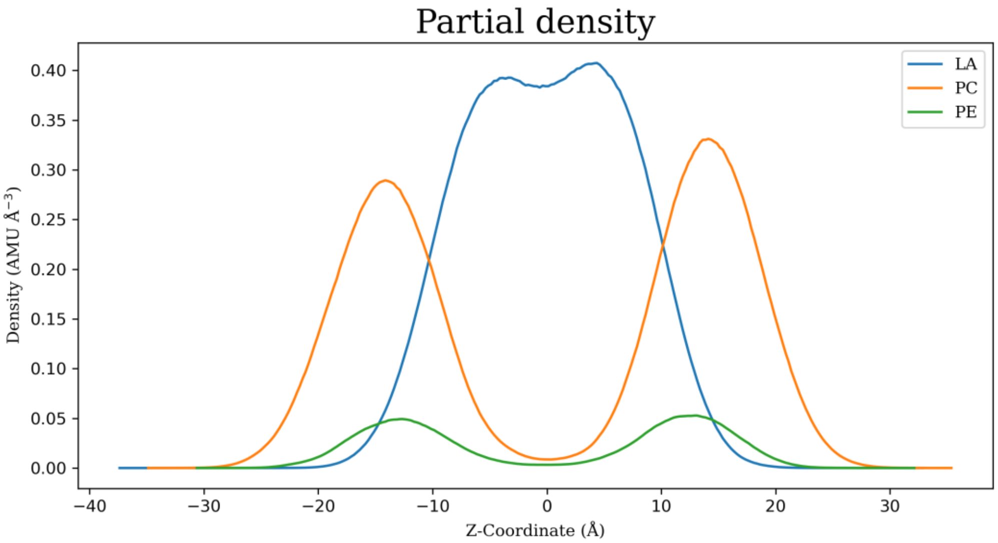

Figure S8. Density calculated for the lipids headgroups (phosphatidylcoline, PC; phosphatidylethanolamine, PE) and for the acyl chains (lauroyl, LA) of the membrane, averaged along the trajectory and the surface area of the inward-facing BdFpn structure with an excess of Fe^{2+} ions.

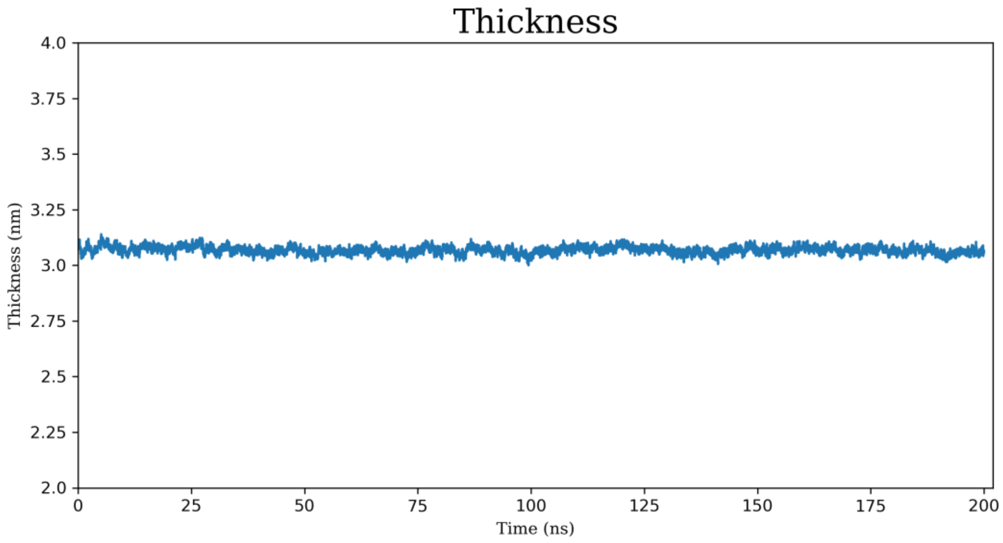

Figure S9. Thickness of the membrane bilayer of the inward-facing BdFpn structure measured along the trajectory.

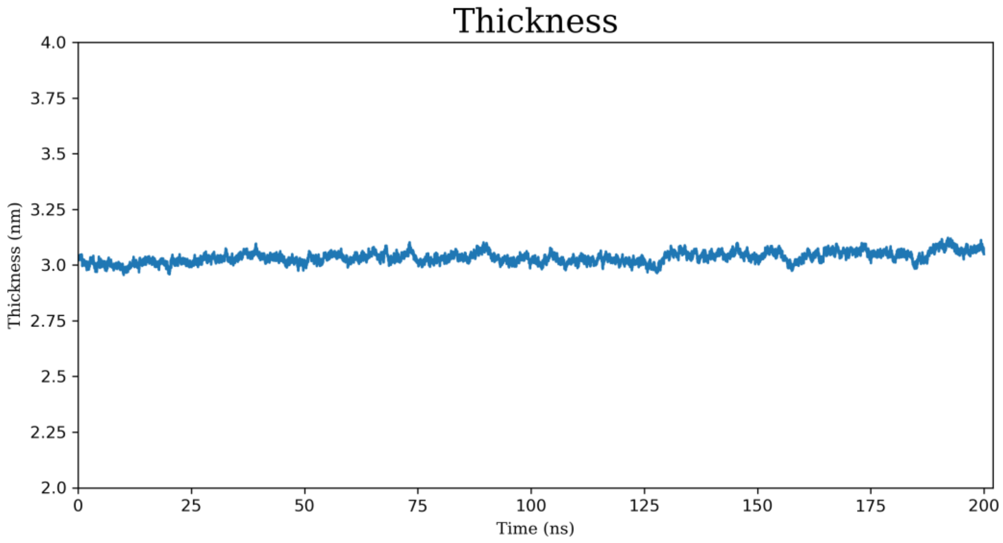

Figure S10. Thickness of the membrane bilayer of the outward-facing BdFpn structure measured along the trajectory.

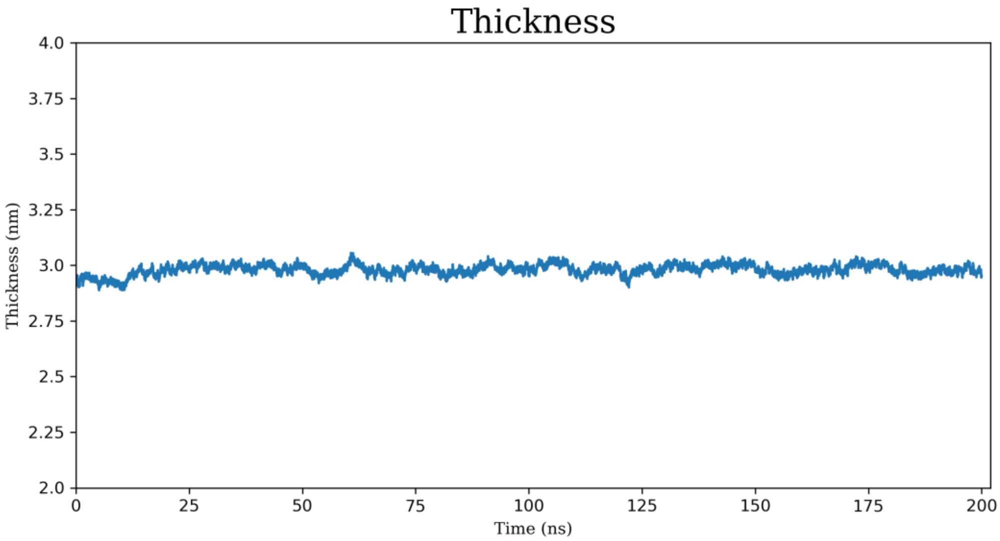

Figure S11. Thickness of the membrane bilayer of the inward-facing BdFpn structure with an excess of Fe^{2+} ions measured along the trajectory.
